# Quality of life and patient satisfaction among individuals with cutaneous leishmaniasis in Syria: a cross-sectional study

**DOI:** 10.1186/s40249-025-01409-2

**Published:** 2026-01-06

**Authors:** Mohamad Yousef Almawaz, Fatima Al-Assi, Eyad Katbi, Abdulrahman Hmidan, Alyaa Kheirbek, Naya Al aeddin, Dana Alshohof, Ahmad Bishr Nasra, Hani Abo Moghdob, Hussein Abdallah

**Affiliations:** 1 Faculty of Medicine, Damascus University, Damascus, 10001 Syria; 2 Faculty of Medicine, University of Aleppo, Aleppo, Syria; 3 Faculty of Medicine, Tishreen University, Latakia, Syria; 4 University Hospital for Dermatology and Venereal Disease, Damascus, Syria

**Keywords:** Cutaneous leishmaniasis, Quality of life, Patient satisfaction, Syria, Neglected tropical diseases

## Abstract

**Background:**

Cutaneous leishmaniasis (CL) is a neglected tropical disease with substantial physical, psychological, and social consequences, particularly in endemic, resource-limited settings. This study assessed the impact of CL on health-related quality of life (HRQoL) and patient satisfaction with care in an endemic region of Syria.

**Methods:**

A cross-sectional study was conducted from May 1 to July 1, 2024 in Damascus University Hospital for Dermatology, Aleppo University Hospital, and two Ministry of Health CL treatment centers in Aleppo, Syria. HRQoL was measured using the dermatology life quality index (DLQI), and patient satisfaction using patient satisfaction questionnaire short form (PSQ-18). Sociodemographic and clinical characteristics were collected. Data were analyzed using SPSS (version 29). Medians and interquartile ranges (IQR) were used to describe non-normally distributed variables. Statistical tests included the Mann–Whitney U, Kruskal–Wallis, and Spearman's rank correlation. A *P*-value < 0.05 was considered significant.

**Results:**

A total of 353 patients (55.9% female; median age 33 years) participated. The median DLQI score showed modest impairment, with the Symptoms and Feelings domain most affected (median 2, IQR 1–3; 87.5% scoring > 0). Female sex was significantly associated with higher Symptoms and Feelings (*P* = 0.018) and Personal Relationships (*P* = 0.020) scores. Head/neck lesions were significantly associated with worse Personal Relationships scores (*P* = 0.014). Satisfaction was generally high, with the highest median scores in Technical Quality (median 16, IQR 14–17) and Accessibility and Convenience (median 14, IQR 12–16). Urban residence was associated with higher General Satisfaction (*P* < 0.001), while number of treatment visits negatively correlated with Accessibility and Convenience (*ρ* = – 0.112, *P* = 0.035).

**Conclusions:**

CL in Syria imposes measurable psychosocial and functional burdens, particularly among women and those with visible lesions. Despite overall high satisfaction with care, disparities related to geography and treatment logistics persist. Addressing psychosocial needs and inequities in care accessibility may improve patient outcomes endemic settings.

## Background

Leishmaniasis is a parasitic disease caused by intracellular protozoa of the genus *Leishmania* [1]*.* Globally, an estimated 700,000 to 1 million new cases occur annually [2]. More than 12 million people are currently infected, and approximately 350 million remain at risk of infection. Leishmaniasis is endemic across several continents, including Asia, Africa, Europe, and the Americas, with a substantial disease burden, particularly in low- and middle-income regions within these areas [1]. The World Health Organization (WHO) classifies leishmaniasis as a neglected tropical disease (NTD) [3].

Leishmaniasis is transmitted through the bite of infected female sandflies; *Phlebotomus* species in the Old World (Asia, Africa, and Europe) and *Lutzomyia* species in the New World (the Americas). Sandfly activity is most intense on warm, calm nights, with low wind speed. Leishmaniasis predominantly affects impoverished communities in low-income countries, where weak healthcare infrastructure, limited funding for control programs, and low pharmaceutical investment hinder effective management [2].

Leishmaniasis manifests in three main clinical forms: localized cutaneous leishmaniasis (CL) presenting mainly with chronic skin ulcers, muco-cutaneous leishmaniasis (MCL) involving mucosal and cartilaginous tissues, and visceral leishmaniasis (VL) affecting internal organs such as the liver, spleen, and bone marrow. Both MCL and VL can be lethal if left untreated [3].

CL, accounting for an estimated 600,000 to 1 million new cases annually, is the most prevalent form of leishmaniasis worldwide. CL is associated with chronic ulcers that may result in permanent disfiguring scars and can be accompanied by satellite lesions or nodular lymphangitis [3]. CL has been endemic in Syria for centuries and is commonly referred to as the “Aleppo boil”. Historical WHO records indicate around 14,000 new cases per year between 1994 and 2000, with incidence rising to over 27,000 by 2010 [4]. Conflict and mass displacement in recent years have intensified outbreaks, largely due to overcrowding and disruption of healthcare services. Although CL is rarely fatal, the disease leaves disfiguring scars, and can profoundly reduce quality of life, particularly when lesions persist for extended periods [5]. Cutaneous leishmaniasis also carries a substantial psychosocial burden, including stigma, social isolation, reduced self-esteem, and emotional distress, particularly among women and individuals with visible lesions. Recent reviews and qualitative studies have emphasized how CL-related disfigurement affects interpersonal relationships and mental well-being in endemic regions [6].

Few studies have reviewed the impact of CL in Syria, although there have been numerous studies showing the impact of Syrian refugees on the spread of disease in many neighboring countries such as Lebanon and Turkey [7, 8]. A systematic review and meta-analysis of CL prevalence in the Middle East reported that Syria has the highest estimated pooled prevalence in the region at 39% (95% CI 37–42%). This high burden is largely attributed to the ongoing war and humanitarian crisis, which is associated with a considerable increase in prevalence, the deterioration of healthcare infrastructure, and the mass displacement of populations. Historically, CL in Syria was largely confined to specific foci such as Aleppo and Damascus [9].

In this study, we aimed to assess the impact of cutaneous leishmaniasis on patients’ quality of life and satisfaction with treatment in Syria by examining both the physical and socio-emotional dimensions of the disease, with the objective of informing evidence-based strategies to improve patient outcomes and reduce its burden in Syria and comparable regions.

## Methods

### Study design

A cross-sectional design was chosen as it allows for the collection of data at a single point in time. The study was conducted over a two-month period, from May 1, 2024, to July 1, 2024, at Damascus University Hospital for Dermatology, Aleppo University Hospital, and two Ministry of Health cutaneous leishmaniasis (CL) treatment centers in Aleppo, Syria. A non-probability convenience sampling strategy was used. All adults with confirmed cutaneous leishmaniasis who attended the participating dermatology centers during the study period and met the inclusion criteria were consecutively invited to participate. Random sampling was not feasible due to variable daily patient flow and logistical constraints at the clinical sites. The study was non-interventional, meaning that participants did not receive any medical treatment or advice as part of the research.

### Participants and data collection

#### Participants

Eligible participants were adults aged ≥ 18 years with a confirmed diagnosis of cutaneous leishmaniasis. Diagnosis was established through microscopic identification of amastigotes in skin smears, supported by clinical assessment according to local practice.

Participants were excluded if they declined to provide informed consent, had significant neurological or psychiatric conditions impairing their ability to respond, or completed the questionnaire incompletely or with missing mandatory items.

A total of 378 eligible patients were approached; 353 consented and completed the questionnaire.

#### Data collection process

Data were collected using face-to-face interviewer-assisted interviews, conducted by trained medical investigators to ensure consistency and to accommodate participants with varying literacy levels. Participation was anonymous, with each respondent completing the questionnaire only once. No personal identifiers were recorded. The structured questionnaire included sociodemographic characteristics, clinical features, the DLQI scale, and the Patient Satisfaction Questionnaire Short Form (PSQ-18).

Demographic information included age, sex, marital status, province, residence, educational level, employment status, economic status, and medical history.

Clinical characteristics included the number of lesions, ulceration present, duration of lesions, history of relapse, location of the lesions and treatment. No formal clinical classification scheme was applied; diagnosis was based on microscopic identification of amastigotes supplemented by clinical assessment according to local practice.

#### Sample size

The minimum sample size required to estimate proportions with acceptable precision was calculated based on a 95% confidence level and a 5% margin of error. Because no prior estimate for the main outcome in this specific population was available, we used a conservative response distribution of 50% (which maximizes the required sample size). Using the standard sample size formula for proportions

$$ \text{n}=\frac{{Z}^{2} pq}{{d}^{2}}$$

With (*Z* = 1.96, *p* = 0.5, *d* = 0.05), the theoretical sample-size requirement is 384. During the two-month recruitment period, 378 eligible adults with confirmed CL were approached across the participating centers. Of these, 25 individuals declined participation, resulting in a non-response rate of 6.6%. A total of 353 participants completed the questionnaire and were included in the final analysis. With this achieved sample, the precision remains acceptable, yielding a margin of error of approximately 5.2% for proportions around 50%, making the sample adequate for this cross-sectional descriptive study.

#### Measures

##### Quality of life assessment

Health-related quality of life was assessed using the Dermatology Life Quality Index (DLQI), which includes 10 items across six domains: symptoms and feelings, daily activities, leisure, work/school, personal relationships, and treatment. Each item is scored from 0 to 3, yielding a total score from 0 to 30, where higher scores indicate greater impairment.

The questionnaire was administered using the official Arabic version validated by Cardiff University. The translation process followed a forward–backward procedure to ensure conceptual and linguistic equivalence. Internal consistency for the Arabic version in our pilot testing (*n* = 30) showed a Cronbach’s alpha of 0.85, indicating good reliability [10].

##### Patient satisfaction assessment

Patient satisfaction was measured using the PSQ-18, which evaluates seven domains: General Satisfaction, Technical Quality, Interpersonal Manner, Communication, Financial Aspects, Time Spent with Doctor, and Accessibility and Convenience. Items are scored on a 5-point Likert scale (1 = strongly disagree to 5 = strongly agree). Negatively worded items were reverse-scored so that higher scores consistently reflected higher satisfaction. Each domain score was calculated as the mean of its corresponding items [11].

##### Statistical analysis

Data were analyzed using IBM SPSS Statistics (version 29; IBM Corp., Armonk, NY, USA). Descriptive statistics were used to summarize all variables: medians and interquartile ranges (IQR) for continuous variables, and frequencies and percentages for categorical variables. Internal consistency of the DLQI and PSQ-18 subscales was assessed using Cronbach’s alpha. Given the non-normal distribution of most variables, non-parametric methods were used. Mann–Whitney U tests compared two-group variables (e.g., sex, marital status). Kruskal–Wallis tests compared variables with ≥ 3 groups (e.g., education, number of lesions). Spearman’s rank correlation evaluated relationships between continuous variables (e.g., age, number of treatment visits). A *P*-value < 0.05 was considered statistically significant.

##### Ethical considerations

Ethical approval was obtained from the Ethical Committee of Damascus University, Faculty of Medicine (MD-261124–360). Written informed consent was obtained from all participants prior to data collection. Confidentiality was ensured by anonymizing questionnaires and excluding any identifying information. All study procedures adhered to the Declaration of Helsinki guidelines for ethical research involving human participants.

## Results

### Participant characteristics

The median age was 33 years (IQR: 23–48). Most participants were female (55.9%), married (65.3%), and residents of Aleppo (74.3%). Over half had primary education or less (60.5%), 54.8% were unemployed, and 65.5% reported moderate economic status (Table 1).

**Table 1:** Demographic characteristics of patients with cutaneous leishmaniasis (*n* = 353)

Variable		Frequency		Percentage		Median (Interquartile range)
Age		–		–		33 (23–48)
*Sex*						
Male		156		44.1		–
Female		197		55.9		–
*Marital Status*						
Single^1^		123		34.7		–
Married		230		65.3		–
*Province*						
Aleppo		262		74.3		–
Rif Dimashq		44		12.4		–
Damascus		32		9		–
Others^2^		15		4.3		–
*Residence*						
Urban		225		63.8		–
Rural		128		36.2		–
*Educational Level*						
Primary school or lower		213		60.5		–
Secondary school		82		23.2		–
Higher education		58		16.4		–
*Employment Status*						
Employed^3^		146		41.2		–
Unemployed		193		54.8		–
Retired		14		4		–
*Economic Status*						
Poor		84		23.7		–
Moderate		231		65.5		–
Good		38		10.7		–
*Medical History*						
Yes^4^		76		21.5		–
No		277		78.5		–

1. Includes: Single, Widowed, and Divorced individuals

2. Includes: Idlib (0.8%), Al-Hasakah (0.3%), Quneitra (0.8%), Deir al-Zor (0.6%), Latakia (0.8%), Homs (0.3%), and Daraa (0.6%)

3. Includes: Employed and Freelance/Self-Employed individuals

4. Includes: Hypertension (9%), Diabetes mellitus (3.1%), Hypertension and diabetes mellitus (3.4%), and Others (6%)

### Clinical characteristics of cutaneous leishmaniasis

Most patients had a single lesion (41.0%) or ≥ 4 lesions (22.9%). Ulceration was present in 56.5% of cases. Lesion duration was most often 2–5 months (50.6%). One in five reported relapse (20.6%). Upper limbs were the most common location (36.2%). The majority were treated topically (77.7%), while 21.5% received systemic treatment (Table 2).

**Table 2:** Clinical characteristics of patients with cutaneous leishmaniasis (*n* = 353)

Variable	Frequency	Percentage
*Number of lesions*		
1	144	41.0
2	77	21.8
3	51	14.4
≥ 4	81	22.9
*Ulceration Present*		
Yes	199	56.5
No	154	43.5
*Duration of Lesions*		
< 2 months	47	13.3
2–5 months	178	50.6
6–10 months	54	15.3
> 10 months	74	20.9
*History of Relapse*		
Yes	73	20.6
No	280	79.4
*Location of the lesions*		
Upper limbs	127	36.2
Lower limbs	80	22.6
Head/Neck	54	15.3
Upper and lower limbs	53	15.0
Mixed/Generalized^1^	39	11.0
*Treatment*		
No treatment	3	0.8
Systemic	76	21.5
Topical	274	77.7

1. Three or more regions involved, or any case involving the trunk

### Quality of life scores (DLQI)

Median scores indicated modest quality-of-life impairment. Symptoms and Feelings was most affected (median 2, IQR 1–3; 87.5% scoring > 0). Daily Activities followed (median 1, IQR 0–2; 65.7% > 0). Other domains—Leisure, Work/School, Personal Relationships, and Treatment—had medians ≤ 1, with fewer than half of patients scoring above 0 in most domains (Table 3).

**Table 3:** DLQI domain scores in patients with cutaneous leishmaniasis

DLQI Domain	Median (IQR)	Range	% > 0
Symptoms and Feelings	2 (1–3)	0–6	87.5%
Daily Activities	1 (0–2)	0–6	65.7%
Leisure	0 (0–2)	0–6	49.9%
Work/School	0 (0–1)	0–3	33.4%
Personal Relationships	0 (0–1)	0–6	27.5%
Treatment	0 (0–1)	0–3	43.1%

*DLQI* Dermatology Life Quality Index; *IQR* Interquartile range

### Factors associated with quality of life

Female sex was significantly associated with greater impairment in Symptoms & Feelings (*P* = 0.018) and Personal Relationships (*P* = 0.020). Age was weakly negatively correlated with the Treatment domain (*ρ* = –0.113, *P* = 0.033). Clinically, the number of lesions was associated with the Treatment domain (*P* = 0.036), with patients having three lesions reporting the highest treatment-related impairment, while those with one or ≥ 4 lesions reported the lowest. Lesion location was associated with Personal Relationships (*P* = 0.014; highest in head/neck lesions), lesion duration with Leisure (*P* = 0.045; greatest impact in < 2 months), and relapse history with Work/School (*P* = 0.049; unexpectedly higher in non-relapse patients). No significant associations were found with marital status, residence, education, employment, economic status, medical history, ulceration, treatment type, or treatment times (Table 4).

**Table 4:** Association between demographic and clinical characteristics and DLQI domains

DLQI Domain	Age (*ρ, P*)	Sex (*P*)	Number of Lesions (*P*)	Lesion Location (*P*)	Duration (*P*)	Relapse (*P*)
Symptoms and Feelings	− 0.025, 0.636	0.018^*^	0.923	0.458	0.860	0.199
Daily Activities	− 0.084, 0.117	0.154	0.624	0.976	0.191	0.183
Leisure	− 0.065, 0.223	0.689	0.758	0.996	0.045*	0.452
Work/School	− 0.035, 0.512	0.944	0.722	0.866	0.093	0.049*
Personal Relationships	− 0.010, 0.849	0.020^*^	0.089	0.014*	0.608	0.431
Treatment	− 0.113, 0.033*	0.331	0.036*	0.846	0.372	0.373

Note: Values in bold indicate statistically significant results (*P* < 0.05). *DLQI* Dermatology Life Quality Index

### Patient satisfaction scores (PSQ-18)

Satisfaction levels were generally high. Technical Quality (median 16, IQR 14–17) and Access/Convenience (median 14, IQR 12–16) had the highest scores. General Satisfaction, Interpersonal Matters, Communication, Financial Aspects, and Time with Doctor all had medians of 8, with IQR values between 6 and 9 (Table 5).

**Table 5:** Patient Satisfaction Scale (PSQ-18) scores among patients with cutaneous leishmaniasis

Subscale	Median (Interquartile range)	Range
General satisfaction	8 (6–8)	2–10
Interpersonal manner	8 (7–9)	2–10
Technical quality	16 (14–17)	4–20
Communication	8 (6–9)	2–10
Financial aspects	8 (6–9)	2–10
Time spent with doctor	8 (6–8)	2–10
Accessibility and convenience	14 (12–16)	4–20

### Factors associated with patient satisfaction

Several demographic and clinical variables were significantly associated with satisfaction. Females reported higher General Satisfaction (*P* = 0.004), while males scored higher in Interpersonal Matters (*P* = 0.005) and Financial Aspects (*P* = 0.033). Province was associated with General Satisfaction (*P* = 0.002; highest in Aleppo, lowest in Rif Dimashq), Time with Doctor (*P* = 0.002; highest in Damascus, lowest in Aleppo), and Access and Convenience (*P* = 0.012; highest in Damascus, lowest in other provinces). Urban residents had higher General Satisfaction than rural residents (*P* < 0.001). Retired patients reported the highest scores in Interpersonal Matters, Communication, and Time with Doctor. Higher income was associated with greater satisfaction in Financial Aspects (*P* = 0.011). Clinically, the number of lesions was associated with General Satisfaction (*P* = 0.013; ≥ 3 lesions highest) and showed a borderline effect on Financial Aspects (*P* = 0.050). Relapse was borderline associated with General Satisfaction (*P* = 0.050; higher in non-relapse patients). Treatment type was associated with multiple domains: topical treatment with General Satisfaction (*P* = 0.012), systemic treatment with Interpersonal Matters (*P* = 0.025), and both systemic and topical treatment with higher Technical Quality (*P* = 0.044). Number of treatment times was negatively correlated with Access and Convenience (*ρ* = –0.112, *P* = 0.035). No associations were observed with medical history, ulceration, lesion duration, lesion location, or current treatment type (Table 6).

**Table 6:** Association between demographic and clinical characteristics and patient satisfaction domains

Satisfaction subscale	Sex (*P*)	Residence (*P*)	Province (*P*)	Employment (*P*)	Economic Status (*P*)	Number of Lesions (*P*)	Relapse (*P*)	Treatment Type (*P*)	Treatment Times(*ρ*, *P*)
General satisfaction	0.004^*^	< 0.001^*^	0.002*	0.063	0.062	0.013*	0.050	0.012*	− 0.066, 0.213
Interpersonal manner	0.005^*^	0.646	0.062	0.006*	0.318	0.595	0.341	0.025*	− 0.076, 0.157
Technical quality	0.443	0.198	0.274	0.162	0.535	0.508	0.975	0.044*	− 0.054, 0.310
Communication	0.716	0.063	0.854	0.040*	0.227	0.451	0.664	0.121	− 0.051, 0.337
Financial aspects	0.033^*^	0.295	0.398	0.083	0.011*	0.050	0.499	0.078	− 0.032, 0.547
Time spent with doctor	0.132	0.196	0.002*	0.044*	0.802	0.749	0.985	0.472	− 0.025, 0.639
Accessibility and convenience	0.239	0.650	0.012^*^	0.126	0.373	0.154	0.988	0.818	− 0.112, 0.035^*^

Note: Values in bold indicate statistically significant results (*P* < 0.05)

## Discussion

The impact of CL on individuals is frequently linked to the physical disfigurement resulting from the disease [12]. This study provides a comprehensive assessment of quality of life and patient satisfaction among individuals with CL, a prevalent parasitic disease in Syria, using the dermatology life quality index (DLQI) and PSQ-18. This finding underscores the importance of a patient-centered approach to healthcare, which goes beyond clinical outcomes to address the psychosocial and emotional burdens of the disease [13]. It also highlights key demographic and clinical factors linked to both quality of life and patient satisfaction, contributing important insights for healthcare management and public health planning. Although the study's overall median DLQI scores indicated only a modest impairment in patients' quality of life, a more granular analysis revealed a significant burden in the 'Symptoms and Feelings' domain. This domain had a median score of 2 (IQR: 1–3) and affected nearly all patients in the cohort (87.5%) [14].

Chronic skin conditions like psoriasis and leprosy, which unfortunately still carries a lot of stigma and can lead to permanent deformities, often have a bigger overall impact on quality of life than what was seen in this Syrian CL group. Many studies show that people with moderate or severe psoriasis usually score quite high on the DLQI. This puts them in the moderate to very large effect range (DLQI ≥ 6). One large meta-analysis even found an average DLQI of 10.5 for plaque psoriasis, mostly because of problems in the Symptoms and Feelings and Personal Relationships sections similar to CL, but with a stronger impact [15]. In leprosy, quality of life is heavily influenced by visible deformities and social stigma. Reported mean DLQI scores are usually between 6.1 and 8.4, which fits the moderate effect category. In groups with severe physical disability or lepra reactions, the scores can go much higher, reflecting major difficulties in Daily Activities and Personal Relationships because of disfigurement and isolation [16].

Compared to the higher DLQI scores often seen in psoriasis, the relatively mild impairment in this Syrian CL cohort suggests that CL lesions, although emotionally distressing, especially in the Symptoms and Feelings domain, may be more localized, shorter-lived, or simply less disabling in daily life than chronic, widespread diseases. This highlights how important it is to consider the psychological and social impact of scarring, not only the physical limitations.

Some studies have shown a greater quality-of-life burden than what appeared in this Syrian sample. For example, a study from Pakistan reported a mean DLQI of 11.83, which falls into the moderate effect range [14]. Another study from Brazil found that 70% of patients had DLQI scores showing at least a moderate impact [17].

The gap between this Syrian study and others reporting bigger effects could be explained by: (1) Disease Stage: Whether patients were in the active, healing, or scarring stage when they filled out the questionnaire. (2) Cultural Factors: Differences in how societies view visible skin disease, stigma, and physical appearance. (3) Access to Care: Patients in this Syrian cohort reported high satisfaction with their care (based on PSQ-18 scores), which might reduce emotional distress and lead to lower DLQI scores compared to places where treatment is harder to access.

Daily Activities showed notable impairment, particularly among women, reflecting the visibility of lesions and associated functional limitations. In contrast, domains such as Work/School and Personal Relationships were less impacted, though head and neck lesions were significantly associated with social relationship difficulties, suggesting that the cosmetic disfigurement associated with CL is a major concern for patients. The study also brought to light a potential gender-based difference in how the disease impacts patients. We found that females reported a greater burden in the 'Symptoms and Feelings' and 'Personal Relationships' domains. This finding resonates with similar research in dermatology, where women often report a higher degree of psychological distress related to skin conditions, suggesting that healthcare providers should be particularly sensitive to these psychosocial factors when treating female patients with CL [18].

In contrast to the DLQI findings, the results from the PSQ-18 show that patients were generally happy with the care they received, especially the technical quality and convenience of the services. This suggests that they have confidence in their providers and can easily access the healthcare system. However, satisfaction was not consistent across the board. We found geographic disparities, with patients in some provinces reporting lower satisfaction. Additionally, unemployed and low-income patients were less satisfied with the financial aspects of their care and the time they spent with their doctors. These inequities highlight the need for public health policies that improve financial accessibility and provide more personalized care to address patient-specific needs.

Our study found that females were more affected than males in symptoms, feelings, and personal relationships, which we attribute to the societal stigma associated with cutaneous leishmaniasis that impacts females more than males. This finding is consistent with a study conducted in Tunisia [19].

Patient satisfaction is an important element in the quality of healthcare services and as it is influenced by many factors such as the patient, medical staff and the infrastructure of the center, it has become difficult to measure patient satisfaction and service quality [20].

The study showed that satisfaction among urban residents was higher than rural residents because of the difficulty of accessing care centers in the city center due to the long duration of treatment, frequent visits, and the additional burden of transportation costs to the city [12].

It should be noted that treatment in Syria is given at no cost, a long time is taken before diagnosis, treatment is prolonged and sometimes the disease returns. The problems mentioned above are mostly due to the neglect of patients and the exhausting nature of the long treatment process. Leishmania severely interfered with the patients' lives, as they had to visit the clinic very often and, on top of that, the disease imposed certain physical limitations that made their daily lives increasingly difficult [21].

The limitations of this study are: first, the main limitation is its reverse causality bias, which is inherent to cross-sectional studies; second, since the participants were adults, the findings may not be applicable to younger populations; third, because this study included only patients of Syrian ancestry, the findings cannot be generalized to other ethnicities; fourth, the analyses were performed using the questionnaire’s cut-off points, which corresponded to the median values of each subscale.

We recommend conducting longitudinal studies to achieve a deeper understanding of the relationship between the variables. Furthermore, the study should be expanded to include broader regions across Syria. The use of a leishmaniasis-specific questionnaire is also advised to obtain more accurate and reliable results.

## Conclusions

Cutaneous leishmaniasis remains a significant public health concern in Syria, exerting a measurable impact on patients’ quality of life, with the greatest burden observed in the symptoms and feelings and daily activities domains. Female patients and those with lesions in visible areas, particularly the head and neck, reported greater psychosocial distress, underscoring the need for gender-sensitive and stigma-aware clinical approaches.

Despite these challenges, overall patient satisfaction with care was high, particularly regarding technical quality and accessibility. However, disparities linked to geographic location, socioeconomic status, and treatment logistics highlight persistent inequities in service delivery. Addressing these gaps could further optimize outcomes, through improved outreach to rural areas, enhanced patient–provider communication, and targeted psychosocial support.

Our findings emphasize that effective CL management in endemic, resource-limited settings must extend beyond clinical cure to encompass the social, emotional, and practical dimensions of patient well-being. Future longitudinal and multi-regional studies, ideally incorporating disease-specific quality of life tools, are warranted to guide tailored interventions and strengthen health system responsiveness to this neglected tropical disease.

## Data Availability

All data generated or analysed during this study are included in this published article.

## Abbreviations

CL: Cutaneous leishmaniasis
HRQoL: Health-related quality of life
DLQI: Dermatology Life Quality Index
PSQ-18: Patient Satisfaction Questionnaire Short Form
IQR: Interquartile range
NTD: Neglected tropical disease
WHO: World Health Organization
MCL: Muco-Cutaneous Leishmaniasis
VL: Visceral leishmaniasis

## Declarations

### Content for publication

During the preparation of this work, the authors used ChatGPT-4 by OpenAI to improve readability and language clarity. After using this tool, the authors reviewed and edited the content as needed and take full responsibility for the content of the publication.
